# Current clinical framework on nitric oxide role in periodontal disease and blood pressure

**DOI:** 10.1007/s00784-024-05913-x

**Published:** 2024-09-12

**Authors:** Leonel Lima, Sara Gaspar, Bárbara S. Rocha, Ricardo Alves, M. Gabriela Almeida

**Affiliations:** 1 Egas Moniz Center for Interdisciplinary Research (CiiEM), Egas Moniz School of Health & Science, Monte da Caparica, Almada, Portugal; 2 UCIBIO/i4HB– Applied Molecular Biosciences Unit, NOVA School of Science and Technology, NOVA University of Lisbon, Caparica, Portugal; 3 Faculty of Pharmacy and Center for Neuroscience and Cell Biology, University of Coimbra, Coimbra, Portugal

**Keywords:** Nitric oxide, Nitrite, Nitrate, Periodontal disease, Periodontitis, Oral microbiome, Diet, Hypertension

## Abstract

**Objectives:**

In this review, we explored potential associations between NO and its derivatives, nitrite and nitrate, with periodontal and cardiovascular diseases, with special emphasis on the former. By providing a state-of-the-art and integrative understanding of this topic, we aimed to shed light on the potential role of these three nitrogen oxides in the periodontitis-hypertension nexus, identify knowledge gaps, and point out critical aspects of the experimental methodologies.

**Materials and methods:**

A comprehensive literature review was conducted on human salivary and plasma concentrations of nitrate and nitrite, and their impact on periodontal and cardiovascular health.

**Results:**

A nitrate-rich diet increases nitrate and nitrite levels in saliva and plasma, promoting oral health by favorably altering the oral microbiome. Chlorhexidine (CHX) mouthrinses disrupt the nitrate-nitrite-NO pathway, reducing NO bioavailability, and potentially affecting blood pressure. This is because CHX eliminates nitrate-reducing bacteria, which are essential for NO production. Although endogenous NO production may be insufficient, the nitrate-nitrite-NO pathway plays a critical role in maintaining appropriate endothelial function, which is balanced by the microbiome and dietary nitrate intake. Dietary nitrate supplementation may lead to beneficial changes in the oral microbiome, thereby increasing the NO bioavailability. However, NO bioavailability can be compromised by reactive oxygen species (ROS) and the uncoupling of endothelial nitric oxide synthase (eNOS), leading to further ROS generation and creating a detrimental cycle. Studies on NO and periodontal disease have shown increased nitrite concentrations in patients with periodontal disease, although these studies have some methodological limitations. In terms of blood pressure, literature suggests that CHX mouthrinses may reduce the capacity of nitrate-reducing bacteria, potentially leading to an increase in blood pressure.

**Conclusions:**

Several studies have suggested an association between NO levels and the development of cardiovascular and periodontal diseases. However, the exact mechanisms linking these diseases remains to be fully elucidated.

**Clinical relevance:**

Nitric oxide (NO) is a signaling molecule that plays a crucial role in several physiological processes such as vascular homeostasis, inflammation, immune cell activity, and pathologies such as hypertension and periodontitis.

## Background

Nitric oxide (NO) is a signaling molecule that plays key roles in various physiological processes in mammals, such as neurotransmission, vascular homeostasis, cytoprotection after ischemic insult, inhibition of platelet aggregation, and host defense mechanisms. NO can be endogenously or exogenously produced in the human body. Endogenous production of NO occurs via the action of nitric oxide synthase (NOS). The exogenous production of NO can result from the intake of nitrate (NO_3_^−^) and nitrite (NO_2_^−^) from the diet, which are then reduced to NO through a series of enzymatic or non-enzymatic reactions. As mentioned above, NO is endogenously produced by NOS isoforms, namely neuronal NOS (nNOS), endothelial NOS (eNOS), and inducible NOS (iNOS). In this process, *L*-arginine is oxidized into *L*-citrulline and NO in the presence of oxygen. ^•^NO is a reactive nitrogen oxide species (RNOS) and it is oxidized to the stable ions, nitrite (NO_2_^−^) and nitrate (NO_3_^−^). Its stability varies according to the environment in which it is found. For instance, when exposed to oxygen in tissues, its reaction rate is relatively slow; however, it reacts quickly with ceruloplasmin in plasma and with the Fe (II)-hemoglobin group in blood, being reduced into the oxyanions nitrite and nitrate [1].

Both nNOS and eNOS are constitutively expressed, whereas iNOS is mainly produced by the immune and inflammatory responses. The major source of NO is eNOS, which regulates the systemic vasodilator tone and blood pressure [1–4]. A perturbation in eNOS activity can cause NO insufficiency, which adversely affects cardiovascular health [3, 5]. However, numerous studies have shown that, under oxygen-limiting conditions, NO can be alternatively produced through the reduction of nitrite by several proteins, enzymes, and non-enzymatic systems. These pathways are enhanced during hypoxia and acidosis, serving as backup storage of NO to complement the *L*-arginine-oxygen-NOS route in blood pressure control. So, the bioactivation of nitrite into NO is now recognized as having an important role in hypoxic vasoregulation [1, 6–8], placing nitrite at the forefront of ‘NO biology’. Dietary nitrate also mediates these effects via its conversion to nitrite in the oral cavity by commensal bacteria [4, 8, 9]. Once nitrite-enriched saliva is swallowed, it is absorbed by the upper gastrointestinal tract leading to a rise in circulatory levels [10–12]. The role of the nitrate-nitrite-nitric oxide axis in the control of the cardiovascular system is thus a hot research topic, and the potential therapeutic effects of nitrate/nitrite supplementation in hypertension and other cardiovascular diseases (CVDs) have been the subject of numerous clinical trials [13].

Due to the important role that oral bacteria play in the nitrogen cycle in humans, for this to function correctly, the microbiome must be balanced. For example, in situations of altered systemic oxygen availability, changes in the abundance of nitrogen oxides, such as nitrate (NO_3_^−^), nitrite (NO_2_^−^), and NO, can occur, leading to dysbiosis of the microbiome and vice versa [14, 15]. Periodontal disease (PD), for instance, is characterized by disruption of the oral microbiome and has been shown to affect the cardiovascular system [16]. In the other hand, some studies have correlated periodontitis and its clinical markers, such as pocket depth and clinical attachment loss, with salivary levels of NO_2_^−^ and NO_3_^−^ [17]. Recently, this topic has been investigated to understand how diseases of the oral cavity, such as periodontitis, can affect the *N*-cycle in humans and their repercussions on systemic health [16]. Therefore, characterization of the oral microbiome is important not only in defining the health status of the oral cavity but also in understanding and systemic health [18].

Considering the role of NO in vascular homeostasis and the impact of the oral microbiome in the human *N*-cycle and PD, and possibly in hypertension, this paper aims to provide an updated review of the periodontal-cardiovascular disease nexus and its relationship with nitric oxide and its metabolites. To this end, this manuscript covers the most recent studies evaluating human salivary and plasma concentrations of nitrate and nitrite and their impact on periodontal and cardiovascular health. We will also evaluate the methodologies chosen and the results obtained and identify gaps in knowledge and future perspectives. An electronic search was conducted using three databases (PubMed, Web of Science, and Scopus) to identify and categorize the results of recent clinical studies in this area. There were no time restrictions, and only English-language papers were selected. The bibliography of existing systematic reviews on the topic was assessed manually to include articles not covered by the electronic search to avoid losing relevant information. The following sections are organized into several connected topics, together with an overview of the current clinical knowledge on the role of NO in periodontal disease and blood pressure.

## Nitric oxide and periodontal disease

### Periodontal disease etiopathology and microbial dysbiosis

According to the World Health Organization, PD affects approximately 19% of the world’s population aged greater than 15 years [19]. Moreover, the Global Burden of Disease Study, published in 2013, indicated that periodontitis was the sixth most prevalent disease in 2010 [20].

**Fig. 1:**
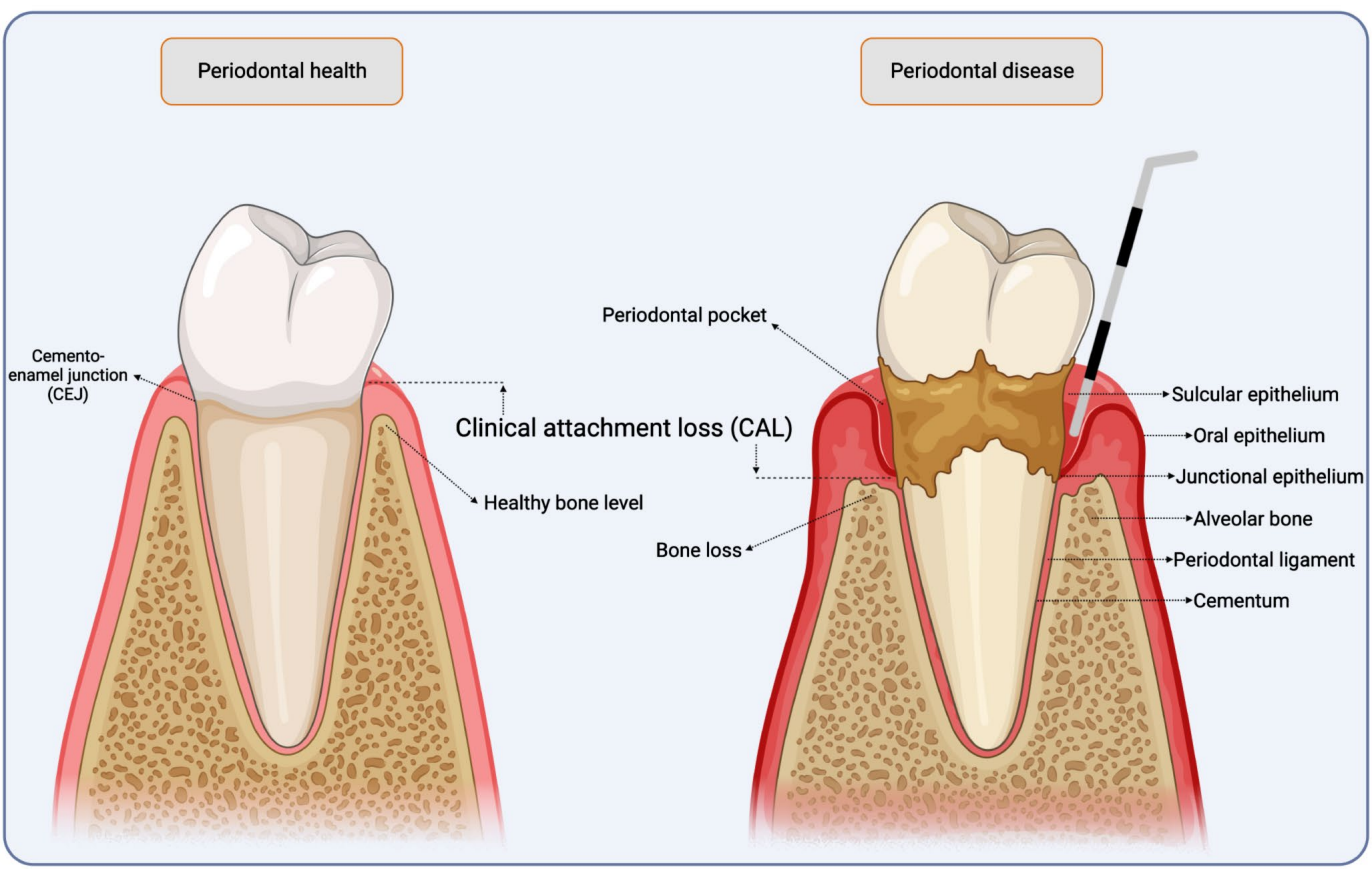
Graphical representation of periodontal health and periodontal disease. Original image. Created on BioRender.com

**Fig. 2:**
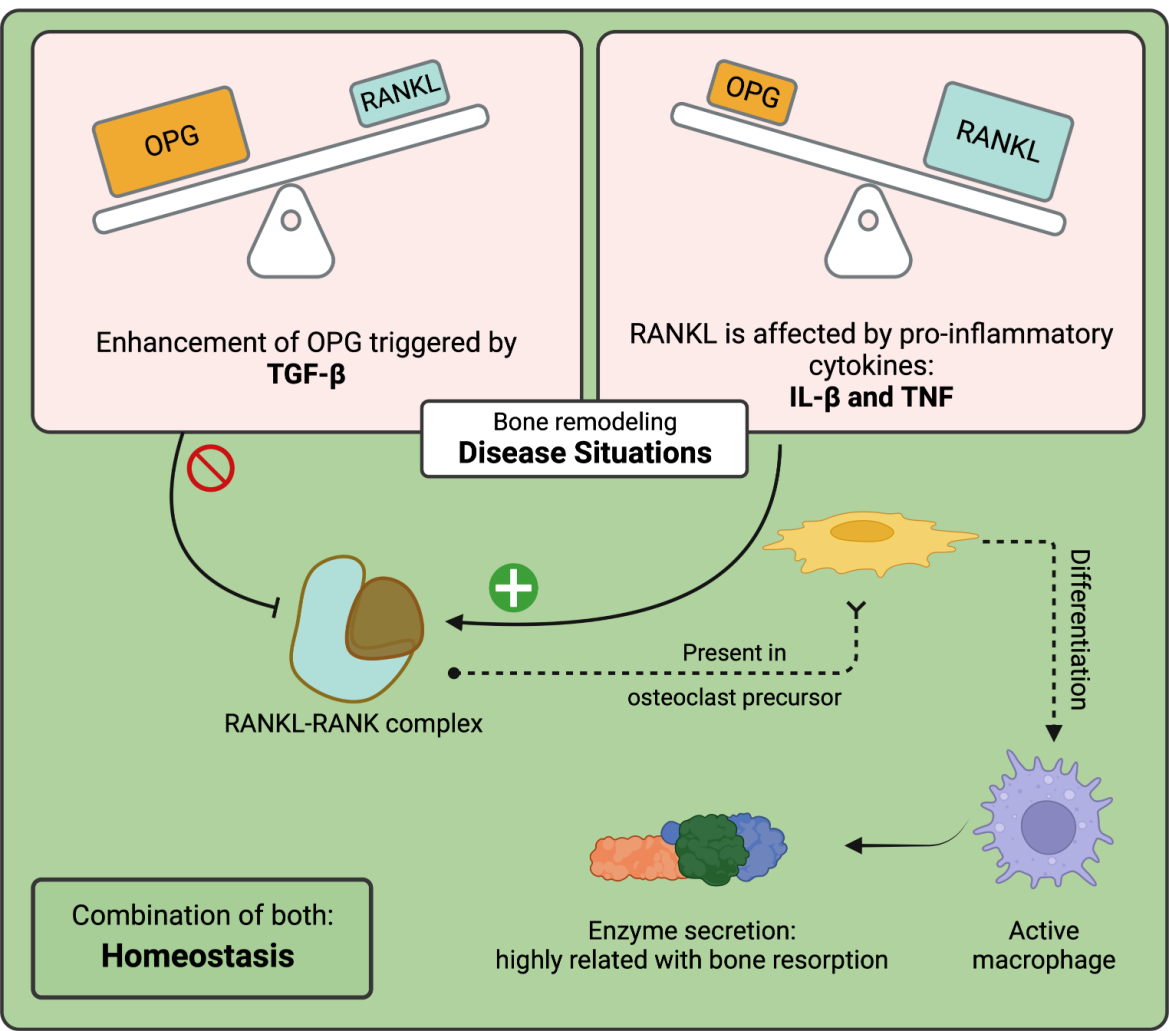
Alveolar bone homeostasis: regulation of RANK-RANKL interaction and bone loss [26]. Original image. Created in BioRender.com

Clinically, periodontitis is defined as periodontal attachment loss (Fig. 1) due to an aberrant interaction between the oral microbiome and the host immune system [21, 22]. Bacterial plaque deposition leads to an inflammatory response that releases pro-inflammatory cytokines, such as IL-1β and TNF, which stimulate the synthesis of the receptor activator of nuclear factor kappa ligand (RANKL). Subsequently, this protein binds to the receptor activator of nuclear factor kappa (RANK) on osteoclasts and activates macrophage-like cells that promote bone resorption. In contrast, osteoprogeterin (OPG) acts as a protector against excessive bone resorption by binding to RANKL and inhibiting its interaction with RANK. The bone marrow OPG/RANKL balance is therefore an important determinant of bone mass under normal and pathological conditions (Fig. 2) [23–26].

The underlying cause of periodontal disease is a dysbiosis of the host oral microbiome. The concept of dysbiosis represents a shift in the microbiome within the gingival sulcus in favor of more periodontal pathogenic species and disfavor of periodontal protective bacterial species, that is, from a state of health to a state of PD [23, 27]. Advances in the study of the etiopathology of periodontal disease suggest that the latter results from a much more complex interaction between the host immune response and microbiota within the periodontal pocket [28]. This contradicts the idea that periodontal disease is only triggered by the presence of a particular pathogenic type of oral anaerobic bacteria like *Porphyromonas gingivalis*, *Treponema denticola*, or *Tannerella forsythia*, usually known by the “red complex” [23, 29, 30].

Several factors may predispose patients to PD. For instance, socioeconomics and demographics [31], genetics [32], and other risk factors such as smoking may influence the severity and aggressiveness of periodontitis [27]. Diabetes mellitus also increases the risk of periodontitis, especially if the disease is uncontrolled. Conversely, periodontal inflammation can hinder the metabolic control of diabetes, thereby enhancing systemic inflammation and insulin resistance [31, 33]. Interestingly, the treatment of PD has been shown to reduce systemic inflammation and improve vascular function, linking the oral cavity and systemic cardiovascular health [24, 34].

As mentioned previously, the balance between osteoclasts (bone resorption) and osteoblasts (bone formation) is very important, as NO may play an important role. For example, when its concentration is elevated, osteoblast function can be reduced as a result of damage to cellular functions caused by oxidative stress [35].

### Nitric oxide and inflammatory response

**Fig. 3:**
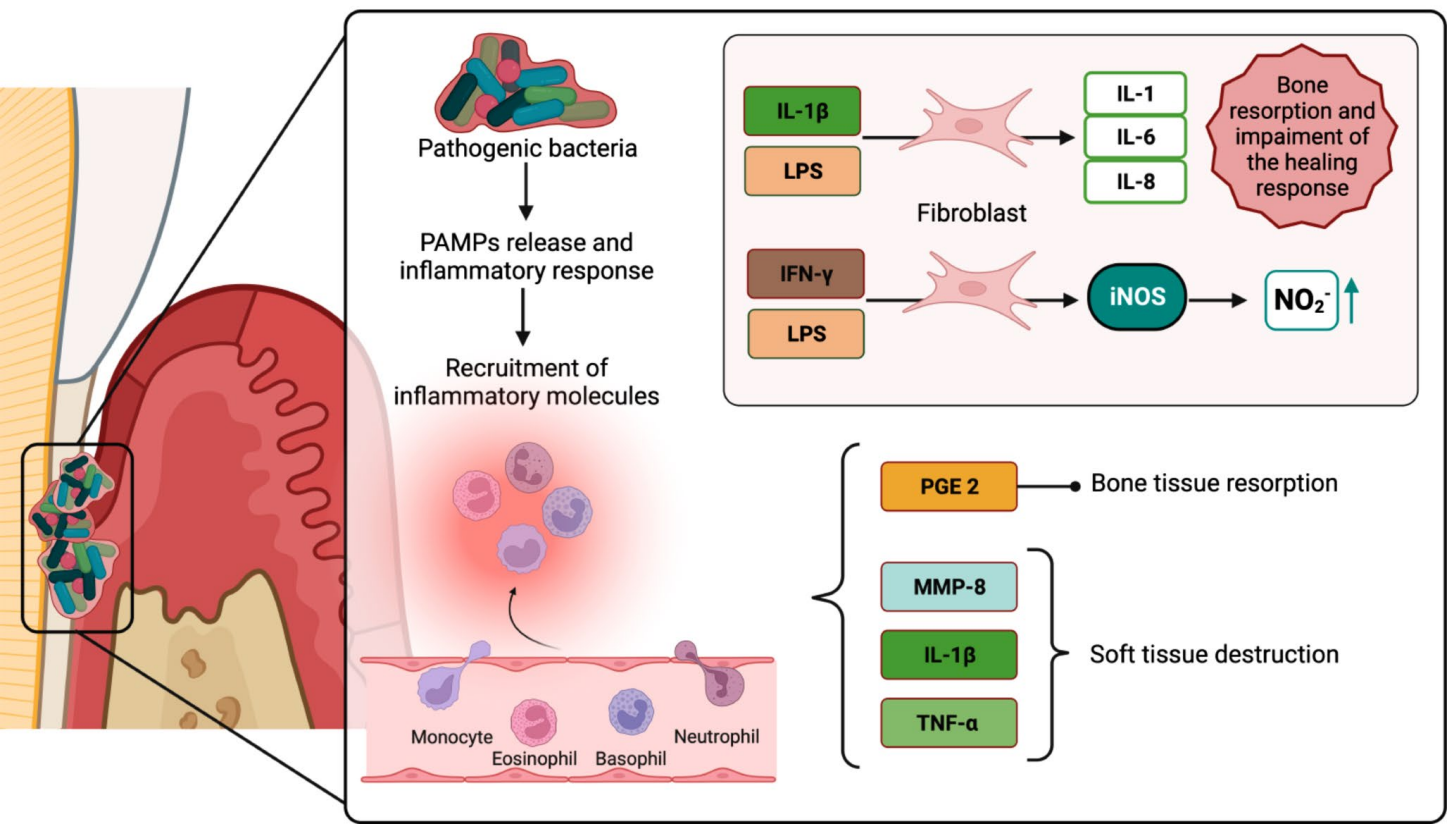
(**A**) Molecular mechanism behind periodontal disease. The bacterial biofilm surrounding the teeth triggers an immune response. The released PAMPs bind to the host cells, and other phagocytic cells and molecules are recruited to the infected local. However, with the cascade switch-off failure and pro-inflammatory macrophage phenotype, the immune response leads to the release of soft tissue destruction and bone tissue resorption factors, contributing to severe periodontal damage and loss of the healing response. (**B**) IL-1β and LPS can induce gingival fibroblasts to express IL-1,6 and 8. IFN-γ and LPS can activate gingival fibroblasts and induce iNOS expression, leading to impaired local immune responses and unregulated bone resorption and formation. Original image. Created in BioRender.com Adapted from [38, 42, 44, 55].

Investigating the role of NO in PD is a top priority because oral bacteria trigger periodontitis-evolving chronic inflammation of the gingival and bone tissues around the tooth [27, 36]. The molecular processes underlying periodontitis have been extensively studied in human and mouse models to understand its mechanisms and to develop more effective treatments for oral diseases [37, 38]. At first, the gingival tissue acts as physical protection by forming an epithelial layer, preventing infections [39]. Bacterial biofilms, consisting of various pathogenic bacteria, activate the first line of defense, which is release into the area neutrophils, macrophages, complements, lymphoid and chemosensory cells, and receptors [40]. As the disease progresses, the enhancement of Reactive Oxygen Species (ROS) production disrupts the balance between the expression of M1 (pro-inflammatory phenotype) and M2 (anti-inflammatory phenotype) macrophages. Consequently, more M1 macrophages are released into the inflamed area. The biofilms also stimulate the release of pathogen-associated molecular patterns (PAMPs) that bind to the host cells recruiting various phagocytic cells and molecules, including again, the M1 macrophages that infiltrate the area. In a typical infection, phagocytosed microorganisms undergo apoptosis, which neutralizes the process. However, in periodontitis, the elimination of apoptotic cells cancels the signal shift from pro- to anti-inflammatory phenotypes, thus failing to switch off the inflammatory cascade, so the inflammation persists [37, 38]. Throughout the process, biomolecules associated with tissue destruction, such as matrix metalloproteinase-8 (MMP8), IL-1β, and TNF-α (interleukins), are released. These molecules stimulate M1 macrophages to secrete pro-inflammatory cytokines contributing to the destruction of tissue [41]. Other molecules that negatively interfere with bone physiological processes and disrupt the balance between osteoblast/osteoclast activities are also released. For instance, prostaglandin PGE2 favors osteoclast activity, resulting in excessive bone resorption [40]. Interestingly, a study carried out on gingival tissue samples from patients with and without PD showed that in inflamed gingival tissue, fibroblasts can mediate bone resorption and iNOS expression. The activation of fibroblasts by interferon-gamma (IFN-𝛾) and lipopolysaccharides (LPS) down-regulates the process of resorption and bone formation (Fig. 3A) [42]. Lipopolysaccharides are a major toxin of Gram-negative bacteria [43]; together with IL-1β, they can induce gingival fibroblasts to produce IL-1,6 and 8, which leads to bone destruction in PD (Fig. 3B) [44, 45]. In parallel, NO is produced by iNOS, which is activated in macrophages during the inflammatory response. The resulting NO is oxidized into NO_2_^−^; however, in vivo, this is a third-order reaction, making the process quite slow [46]. Despite that, the circulating NO_2_^−^ has been associated as a biomarker of several diseases and the salivary levels have been associated with oral diseases such as PD ([47, 48]). However, other factors can contribute to the higher NO_2_^−^ levels observed in salivary fluid, such as the reduction of NO_3_^−^ into NO_2_^−^ [49]. Unfortunately, this important aspect has not been considered in the experimental design and results discussion of many studies [17, 50, 51]. This topic will be discussed in more detail in Sect. 2.4.

Regarding the cardiovascular system, critical health conditions such as hypoxia and lipoprotein augmentation favor the formation of arginases (ARG), which can inhibit the M2 phenotype. This enzyme reduces the *L*-arginine pools, reduces the available substrate for eNOS, and competes with iNOS to reduce NO. Moreover, overexpression of ARG2 in macrophages increases the production of the cytokines TNF-α and IL-6, contributing to PD. Arginases can regulate cellular functions such as inflammation [52]. This metalloproteins can compete with eNOS for *L*-arginine leading to eNOS uncoupling, thereby resulting in a decrease in NO production and an increase of ROS, this changes reflets on endothelial dysfunction, a critical factor of cardiovascular diseases [52]. Uncoupled eNOS reduces the production of NO, thereby decreasing its vascular protective effects while increasing oxidative stress and the production of superoxide anion and hydrogen peroxide. Also, BH_4_ is required for eNOS functioning and an increased presence of ROS may compromise the availability of this co-factor. Thus, a cycle starts, and ROS causes eNOS uncoupling, leading to increased production of ROS [53, 54].

### Nitric oxide, oral microbiome, and dietary intake

Enzymatically produced NO may sometimes be insufficient to maintain its physiological functions, particularly under hypoxic conditions. Therefore, as an alternative route for NO production, the nitrate–nitrite–nitric oxide pathway plays important pathophysiological functions. In this context, recent advances have shown that nitrate-to-nitrite reduction by oral enterobacteria could play a key role, thus linking the oral microflora to blood pressure [4, 56–59]. Hence, it is important to understand how the complex nitrate-nitrite-nitric oxide pathway works and how it is influenced by the oral microbiome and dietary intake of NO_3_^−^ and NO_2_^−^ [12, 54].

**Fig. 4:**
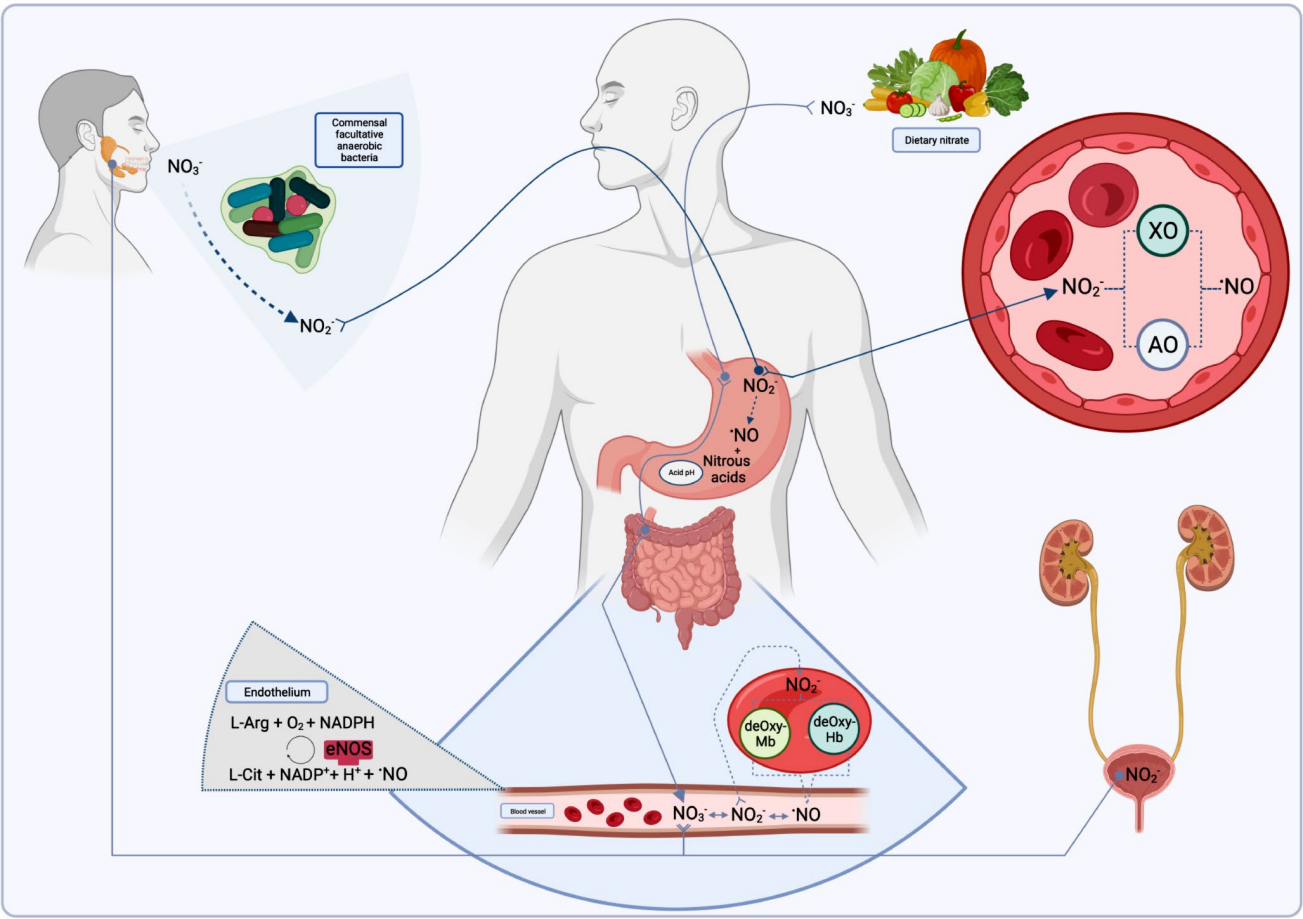
Entero-circulation of nitrate. Legend: NO_3_^-^ (Nitrate); NO_2_^-^ (Nitrite); ^*^NO (Nitric Oxide); XO (Xanthine oxidase); AO (aldehyde oxidase); deOxy-Mb (deoxymyoglobin); deOxy-Hb (deoxyhemoglobin); L-Arg (L-Arginine); L-Cit (L-citruline). Created in BioRender.com [1, 54, 62]

Humans cannot metabolize NO_3_^−^ as they do not have a functional nitrate reductase, making the molecule itself inactive in their bodies. However, bacteria in the human body can convert it into NO_2_^−^, which is a bioactive molecule (Fig. 4) [15, 60, 61]. Briefly, NO_3_^−^ from the diet is absorbed in the small intestine and transported to the salivary glands, where it is stored at a higher concentration compared to plasma. Then, saliva is secreted into the mouth, where commensal facultative anaerobic bacteria present on the surface of the tongue reduce approximately 20% of its NO_3_^−^ content into NO_2_^−^ (Fig. 4). NO_2_^−^ can be further reduced to NO under acidic conditions in the stomach. In addition, NO_2_^−^ may re-enter into circulation and be reduced to NO by xanthine oxidase or aldehyde oxidase [12, 60–63].

According to in vitro assays, part of the *genera*, *Veillonella*, *Actinomyces* and *Rothia*, were confirmed as nitrate-reducing bacteria [64]. Some studies have shown that ingestion of NO_3_^−^ leads to changes in oral microbiota composition. After consuming NO_3_^−^, there is an increase in the abundance of the genera *Rothia* and *Neisseria* at the salivary level. These genera are typically more abundant in subgingival plaques of periodontally healthy patients. Whereas *Veillonella*, *Actinomyces*, *Prevotella* and *Streptococcus* decrease after NO_3_^−^ intake. Particularly, in the case of *Prevotella*, an increase in its abundance was associated with gingivitis and PD.

Typically, when a disruption occurs, the microbiome shifts and becomes specific, increasing the pathogenicity of the individual species [43, 65]. Therefore, it is suggested that there is a reduction in inflammation after NO_3_^−^ ingestion and that this may be due to a change in subgingival plaque [66]. In other words, nitrates can alter the balance between beneficial and harmful bacteria in the mouth, thereby protecting oral health. NO_2_^−^ produced by nitrate-reducing bacteria can be further systemically reduced to NO [39, 66]. Therefore, oral microflora can influence NO homeostasis, which directly affects human health [14, 15, 39]. The opposite also holds true, i.e., an imbalance in reduction/oxidation within the *N*-cycle can also modulate the microbiome. In the oral environment, NO_3_^−^ is rapidly reduced to NO_2_^−^ by nitrate reductase enzymes derived from the bacteria present on the tongue surface. The resulting NO_2_^−^ is then exposed to an acidic environment formed by the dental plaque around the teeth. The acidification of NO_2_^−^ produces NO and nitrogen dioxide (NO_2_). Some authors have proposed that NO_2_^−^in acidic conditions may have an inhibitory effect on the growth and survival of bacteria associated with periodontal disease, including *Porphyromonas gingivalis*, *Fusobacterium nucleatum*, and *Eikenella corrodens* [67]. Therefore, it is possible that an increase in NO_3_^−^ intake, and thus an increase in NO_2_^−^ salivary levels, may act as a protective mechanism against bacteria associated with periodontal disease. Also, if NO concentration is too low, the saliva’s protective effects against bacteria may be reduced, increasing the periodontal tissues susceptibility [67]. Plus, in conditions of low O_2_, NO_3_^−^ is converted to ammonia, which favors the growth of anaerobic bacteria. However, if the concentration of O_2_ is high, ammonia can be reduced to urea or oxidized to nitrite, stimulating the growth of aerobic bacteria [14]. In summary, the microbiome must be well-balanced to maintain the *N*-cycle regulated, and vice versa [14]. It is important to note that the nitrate-nitrite-NO pathway would be compromised without the role of nitrate-reducing bacteria [39, 66].

### Salivary nitrite and nitrate levels and periodontal disease

Ripetska and collaborators found that salivary levels of NO_2_^−^ were increased in patients with chronic periodontitis and gingivitis. Furthermore, the authors suggested that NO_2_^−^ can act as a biomarker of PD, indicating its stage and activity, as well as its aggressiveness [68]. Another study revealed that salivary levels (stimulated and non-stimulated) of NO were positively correlated with the presence of periodontitis and its clinical markers such as pocket depth and clinical attachment loss. Interestingly, higher levels of NO showed a positive correlation between the group of patients with stage IV periodontitis and the family medical history [69]. This work calls for critical comments regarding the identity of the nitrogen species under study. The authors claimed that they measured NO through the Griess reaction, but this method quantified NO_2_^−^ instead of NO. Moreover, although NO_2_^−^ is a stable metabolite of NO, there are other sources of this ion, such as diet, toothpaste (via nitrate reduction) and drugs, that also contribute to the measured levels. Therefore, direct correlations between NO_2_^−^ and NO could not be established. This is a critical mistake that was also found in other studies and deserves careful attention from the readers. Nevertheless, some other studies on the NO-periodontitis correlation based on the Griess reaction correctly presented their data as “nitrite” concentrations. For example, Sundar et al. compared the levels of salivary NO_2_^−^ in patients with chronic generalized periodontitis with a healthy control group and found that its levels were increased in patients with PD [50]. Table 1 describes the main results of several clinical studies that assessed salivary concentrations of NO, NO_2_^−^ and NO_3_^−^ and periodontal disease.

Contrary to previous studies, Moura et al. found no correlation between the presence or severity of periodontal disease and NO levels. In other words, the levels in the patients with and without periodontitis were similar. Curiously, these researchers found a correlation between endothelial dysfunction and increased levels of NO (*p* = 0.03) in saliva [70]. Once again, although the authors claim that they have measured the NO levels, what they have really measured through the Griess method was NO_2_. So; in the study conditions, there was no correlation between the NO_2_^−^ levels in saliva and PD. The same should be considered when drawing conclusions regarding endothelial dysfunction.

Another clinical study that adopted a different method of quantification of NO_3_^−^ and NO_2_^−^ (ozone-based reductive chemiluminescence) also revealed that the salivary levels of these ions were higher in healthy participants than in periodontal patients. In addition, the NO_3_^−^/NO_2_^−^ concentrations remained practically the same in the latter before and after periodontal treatment [51]. On the other hand, an older study revealed that both salivary NO_3_^−^ and NO_2_^−^ were increased in periodontal patients and that their levels decreased after treatment; herein, the authors hypothesized that the inflammatory condition caused by PD leads the salivary glands to increase NO_3_^−^ secretion in saliva [17]. However, NO_3_^−^ is only secreted by the salivary glands when it is present in the diet; otherwise, the concentration in the acini of the salivary glands would not be high enough.

Because NO has a half-life of just 1.8 ms [71], its endogenous formation is usually assessed indirectly by quantifying its stable metabolite NO2- (the total concentration of NO3-/NO2- can be determined after the (bio)chemical reduction of NO3- to NO2-). Most protocols rely on the Griess method, which is based on a colorimetric reaction, followed by spectrophotometric analysis. These and other methods are susceptible to matrix interference and require sample preparation, not allowing accurate measurement of nitrites in physiological samples (values reported for basal plasma and saliva are quite divergent) [72–75]. Chemiluminescence is the most commonly used method for NO detection owing to its high sensitivity and selectivity. In addition, real-time monitoring of NO levels is possible using this technique. However, ozone-based chemiluminescence requires real expertise to perform the analyses, as well as very specific equipment and extremely rigorous sample preparation. This technique is also applicable to the detection of NO, nitrate, and nitrite. These include electrochemical sensors, fluorescence and others. Electrodes can also be used to monitor NO or its derivatives in real-time [76].

**Table 1:** Clinical studies focusing on nitric oxide and periodontal disease

Author	Group, *n*, age range	Method	Results (for disease)	Main conclusion
Ripetska et al. 2021	HC: 10 (20–40) GP: 12 (20–40)	Griess Reaction Saliva (NM)	↑NO_2_^−^ (*p* < 0.05)	Salivary nitrite increased in patients with periodontitis. Possibly related with activity and aggressiveness.
Toczewska et al. 2020	HC:30 (20–55) P-SIII: 36 (20–55) P-SIV: 24 (29–55)	Griess Reaction Saliva E & NE	↑ NO (NE) (*p* < 0.05) ↑ NO (E) (*p* < 0.05)	Positive interchangeability between salivary NO levels in periodontitis and clinical disease markers.
Sundar et al. 2019	HC: 20 (30–55) GP: 20 (30–55)	Griess Reaction Saliva NE	↑NO_2_^−^ (NE) (*p* < 0.05)	Levels of salivary NO_2_^−^ are increased in patients with chronic generalized periodontitis.
Moura et al. 2017	HC: 23 (29–53) GP: 13 (29–53)	Griess Reaction Saliva NE	↓ NO (NE) (*p* > 0.05)	Individuals with and without CP showed similar for NO. A positive correlation between NO and endothelial dysfunction.
Meschiari et al. 2015	HC: 22 (NM) GP: 16 (NM)	Ozone-based reductive chemiluminescence Saliva E	↓NO_2_^−^ (*p* < 0.05)	Lower nitrite concentration in PD patients. Nitrite concentration virtually the same before and after treatment.
Sanchez et al. 2014	HC: 30 (29–53) GP: 44 (29–53)	Griess Reaction Saliva E & NE	↑NO_3_^−^ ;↑NO_2_^−^ (E; NE) (*p* < 0.05)	Total salivary nitrates and nitrites are increased in saliva from patients with periodontal disease.

Legend: P (periodontitis); HC (healthy control); GP (generalized periodontitis); E (stimulated); NE (non-stimulated); S (stage); NM (not mentioned); NO (nitric oxide); NO_2_^−^ (nitrite); NO_3_^−^ (nitrate)

## Nitric oxide bioavailability and cardiovascular system

Cardiovascular diseases originate from the heart and blood vessel disorders. They are the leading cause of death worldwide (32% of total deaths in 2019) [77]. Among several risk factors for CVD, hypertension (elevated blood pressure) stands out for its prevalence (1.28 billion adults) and potential consequences (heart failure/attack and sudden death) [78, 79]. Thus, early hypertension detection is paramount to public health and offers a chance for intervention before the onset of the disorder. The idea is that it may be possible to prevent the development of the disorder by acting at an earlier stage.

As already mentioned, it has been hypothesized that impaired NO production can lead to cardiovascular dysfunction; nonetheless, NO_3_^−^ and NO_2_^−^ from endogenous sources (NO metabolism) and mostly from exogenous sources (food intake) can be converted back to NO, balancing the circulation of this molecule [80]. Indeed, this alternative source of NO is favored by low concentrations of molecular oxygen and an acidic pH [81]. Under such hypoxic conditions, nitrate reaches the bloodstream via gastrointestinal absorption and can be reduced in red blood cells to produce NO by deoxyhemoglobin [53, 54, 61, 82]. Nitric oxide vasodilatation can also be maintained through NO_2_^−^ reduction.

Oxidative stress may reduce NO bioavailability through uncoupling eNOS by (i) oxidizing their cofactors (e.g., BH_4_) and (ii) oxidative inactivation of NO via its reaction with superoxide (O_2_^−^), thereby producing peroxynitrite (ONOO^−^). These events cause endothelial cell dysfunction, which is the first change in atherosclerosis process [80]. Impairments in NOS activity, such as increased activity, have been shown to be associated with heart failure [53].

### Salivary and plasmatic nitrate/nitrite levels and blood pressure

In this section, we will focus **our attention on several** clinical studies that assessed the correlation between salivary NO_2_^−^ and NO_3_^−^ levels and blood pressure. Table 2 summarizes the main results obtained so far.

Barbadoro and co-workers performed an interesting study to analyse the salivary NO levels and the microbiome of subgingival and supragingival plaque in patients with and without hypertension. They found an increased concentration of salivary NO in hypertensive patients and a higher concentration of bacteria in the supragingival plaque. Importantly, normotensive patients had a higher concentration of *Neisseria sublfava*, a type of bacteria normally found in the oral cavity under healthy conditions. There was also a correlation between salivary NO levels, the consumption of green leafy vegetables (the main source of nitrate), and supragingival *Actynomicetemcomitans* levels. An inverse relationship has been found between NO levels and hypertension [83]. Once again, although the authors referred that they measured the NO levels, what they effectively measured was NO_2_^−^ when using the Griess method. Therefore, these conclusions should be reformulated by replacing NO with NO_2_^−^.

A clinical investigation by Ashworth et al. compared the dietary intake of NO_3_^−^ between vegetarians and omnivores and found that vegetarians consumed 24% more NO_3_^−^ than omnivores; however, this difference was not statistically significant, possibly because of the small sample size. Moreover, both groups showed similar levels of NO_3_^−^/NO_2_^−^ in their saliva and blood and similar oral microbiome profiles following treatment with a placebo. Moreover, the use of an antibacterial mouthwash reduced the NO_2_^−^ levels in the saliva and blood of both groups but did not affect blood pressure significantly; nevertheless, an increase in systolic blood pressure was found in both groups. In addition, after treatment with mouthwash (chlorhexidine 0,2%), nitrate-reducing capacity and salivary pH were reduced. The microbiome analysis revealed a significant decrease of 16,9% (vegetarian group) and 17,4% (omnivore group) in genera composed of nitrate-reducing bacteria, mainly *Prevotella* and *Actinomyces*. On the other hand, the *Rothia* genera increased [84].

Kapil and collaborators evaluated the impact of a seven-day chlorhexidine mouthwash on salivary, plasma, and urinary NO_3_^−^/NO_2_^−^ levels and their correlation with blood pressure and the nitrate reduction capacity of oral bacteria. The results indicated a decrease in the concentration of NO_2_^−^, an increase in the level of NO_3_^−^, and a decrease in the nitrate-reducing capacity of the oral bacteria. Systolic and diastolic blood pressure increases were correlated with a decrease in NO_2_^−^ plasma concentration [85]. A comparable study undertaken by Bondonno and collaborators evaluated the effects of a 3-day CHX rinse and observed a decrease in salivary NO_2_^−^ concentration and an increase in NO_3_^−^, accompanied by a decrease in nitrate-reducing capacity. Regarding blood pressure, there was an increase in systolic blood pressure but no increase in diastolic blood pressure. However, no correlation was found between the variations in the levels of NO_3_^−^/NO_2_^−^ and changes in blood pressure in hypertensive treated patients [86]. Similar results were reported by Sundqvist and collaborators, but no differences in plasmatic and salivary NO_3_^−^/NO_2_^−^ levels or blood pressure were found [87]. It is important to note the variations in dietary criteria and populations among the three last works. For instance, Kapil et al. asked patients not to eat vegetables with a high nitrate content, whereas Bondonno et al. only requested low nitrate consumption at breakfast before the clinical visits, and Sundqvist et al. restricted the consumption to only four types of vegetables with a low nitrate content [85–87].

Other studies have shown the negative effects of CHX mouthwashes. Although its antibacterial properties are useful for controlling gingivitis, its prolonged use has deleterious effects at the systemic level by eliminating bacteria responsible for reducing nitrate to nitrite in the oral cavity. Thus, the bioavailability of NO is thus reduced [88].

Another study by Burleigh et al. evaluated the influence of nitrate supplementation and concluded that after 7 days, the levels of NO_3_^−^ and NO_2_^−^ in saliva and plasma as well as the salivary pH increased, and no differences in vascular function were observed. However, after supplementation, there was a change in the oral flora favoring the taxa associated with oral health, with a decrease in bacteria of the genera *Prevotella*, *Streptococcus*, and *Actinomyces* [89].

**Table 2:** Salivary and plasmatic nitrate/nitrite levels and blood pressure

Reference	Group, *n* (age)	Objective	Method	Results	Main conclusion
Ashworth et al. 2019	Vegetarians, 20 (26) Omnivores, 9 (26)	Dietary intake of NO_3_^−^ between vegetarians and omnivores, salivary and plasma concentrations of NO_3_^−^ and NO_2_^−^ and activity and diversity of oral bacteria.	Control: ultrapure water. Test: CHX 0.2% mouthwash. .NO: Ozone-based chemiluminescence Diet recorder: MicroDiet, Downlee Systems, UK.	Vegetarians: ↓NO_2_^−^↑NO_3_^−^ (*p* < 0.05) ↓ pH. Omnivores: ↓ NO_2_^−^ ↑ NO_3_^−^ (*p* > 0.05) ↓ pH.	NO_2_^−^ decreases in salivary and plasma fluids. Did not induce a significant change in BP.
Burleigth et al. 2019	Healthy patients, 11 (30)	Impact of 7 days of NO_3_^−^ supplementation on tongue bacteria, NO metabolites, salivary pH and BP.	.NO analyzer, Analytix, UK. Diet: food diary	After NO_3_^−^supplementation: ↑NO_3_^−^↑NO_2_^−^ in saliva and plasma ↑ pH ↓ Genera *Prevotella*,* Streptococcus* and *Actinomyces*	Adaptions to the oral microbiome do not affect the salivary NO_2_^−^ or vascular response to beetroot juice.
Barbadoro et al. 2020	Normotensives, 25 Hypertensives, 23 (50–70)	Association of salivary microbiome, green leaf consumption and NO with hypertension.	Griess reaction Diet: food frequency questionnaire	Normotensives: ↑ NO_2_^−^ (*p* = 0.023) ↑ *Streptococcus mutans.* Hypertensives: ↑*Actinomycetemcomitans* – subgingival plaque ↑ *Treponema denticula* – supragingival and prosthetic materials.	Hypertension is inversely correlated with salivary NO.
Kapil et al. 2013	Healthy patients, 19 (23.8)	Reduction of endogenously generated NO_3_^−^ to NO_2_^−^ by oral bacteria contribution to circulating NO_2_^−^ levels and BP regulation in healthy subjects.	Test: CHX 0.2% mouthwash. .NO: Ozone-based reductive chemiluminescence Diet: self-reported dietary diaries.	After 7 days of CHX mouthwash: Salivary and plasmatic levels: ↓NO_2_^−^↑NO_3_^−^. ↓ Bacteria NO_3_^−^ reducing capacity (~ 90%). ↑ systolic (*p* = 0.003) and diastolic (*p* = 0.002) BP.	Oral nitrate-reducing bacteria modulate vascular NO_2_^−^. NO_2_^−^ regulates the blood pressure.
Bondonno et al. 2015	Treated Hypertensive, 15 (64.7)	Assess the effects of 3-day use of antibacterial mouthwash on .NO status and blood pressure in treated hypertensive men and women.	Control: tap water. Test: CHX 1.28 mg/mol mouthwash. Salivary .NO: GC-MS. Plasma .NO: gas-phase-chemiluminescence.	After 3 days of CHX mouthwash: Salivary levels: ↓ NO_2_^−^ (*p* = 0.01) ↑NO_3_^−^ (*p* < 0.001). Plasmatic levels: ↓ NO_2_^−^ (*p* = 0.09) and no difference on NO_3_^−^ (*p* = 0.2). ↓ Bacteria NO_3_^−^ reducing capacity. ↑ systolic (*p* = 0.01) and no increase on diastolic (*p* = 0.4) BP.	Interrupting the NO_3_^−^-NO_2_^−^-.NO pathway with CHX mouthwash slightly raised systolic blood pressure in treated hypertensives.
Sundqvist et al., 2016	Heathy females, 19 (23)	Explore the physiological role of endogenous nitrate recycling to .NO on metabolism and blood pressure.	Control: placebo mouthwash Test: CHX 0.2% mouthwash. .NO: gas-phase-chemiluminescence.	After 3 days of CHX mouthwash: Salivary levels: ↓ NO_2_^−^ (*p* < 0.001) ↑NO_3_^−^ (*p* < 0.01). Plasmatic levels: No significant differences on NO_2_^−^ and NO_3_^−^. ↓ Bacteria NO_3_^−^ reducing capacity. Blood pressure: No significant changes.	No effects on plasma nitrite levels or blood pressure.

Legend: BP (blood pressure); CHX (chlorhexidine); .NO (nitric oxide); NO_2_^−^ (nitrite); NO_3_^−^ (nitrate); GC-MS (gas chromatography-mass spectrophotometry)

## Conclusions and future perspectives

This manuscript revised the current understanding of the association between NO/NO_2_^−^/NO_3_^−^ and periodontitis, highlighting the absence of consensus regarding the correlation between the levels of these nitrogen oxides and the occurrence and progression of periodontal disease.

However, other valid conclusions have been achieved so far. For example, a nitrate-rich diet increases the salivary and circulating of both nitrate and nitrite levels. On the contrary, the reduced nitrate-reducing capacity due, for instance, to chlorohexidine mouthwash, interferes with the nitrate-nitrite-nitric oxide pathway affecting ^*^NO bioavailability.

In conclusion, the intricate interplay between all components of the human *N*-cycle, either in health or disease, should be better described to translate benchtop data into clinically useful practices. For example, future studies aiming to better understand the role of the nitrate-nitrite-nitric oxide pathway in patients with cardiovascular disease should consider factors that could influence NO bioavailability in the oral cavity, such as inflammatory conditions like periodontitis, the type of diet, and the use of mouthwashes.

In addition, it is important to reiterate the lack of robustness of much of what is known because of the inaccurate use of analytical methods that quantify nitrite rather than NO and the lack of a suitable study design. Finally, to achieve robust and reliable conclusions, future transverse studies must prioritize the accurate quantification of the targeted *N*-oxide species.

## Data Availability

No datasets were generated or analysed during the current study.
